# Exploring the Link Between Oral Health Factors and Hearing Impairments in Children: A Cross-Sectional Study

**DOI:** 10.7759/cureus.63650

**Published:** 2024-07-02

**Authors:** Alemar N Ghannam, Louei Nahas, Mayssoon Dashash

**Affiliations:** 1 Pediatric Dentistry, Damascus University, Damascus, SYR; 2 Department of Surgery, Division of Otorhinolaryngology, Syrian Private University, Damascus, SYR

**Keywords:** gingivitis, plaque accumulation, caries, oral health, children, hearing impairments

## Abstract

Background

Hearing-impaired children may face challenges in communication, social interaction, academic performance, and emotional well-being, which can have a notable impact on their overall quality of life. Beyond these challenges, oral health can also be significantly impacted. The relationship between hearing impairment and dental diseases is an intriguing and interconnected aspect of overall well-being that merits attention and exploration. This study aimed to assess the relationship between various oral health factors and hearing impairments.

Methodology

This cross-sectional study involved 90 hearing-impaired children aged 6-12 years. To evaluate the children’s hearing abilities, diagnostic tools such as pure-tone audiometry were employed. To measure dental health, the decayed, missing, and filled teeth (DMFT) and decayed, missing, and filled primary teeth (dmft) indices, as well as plaque index (PI) and gingival index (GI) were calculated. The chi‑square test was used to identify significant differences between genders. Spearman’s test was used to determine the correlation between variables.

Results

The severity of hearing impairment varied, with 5.6% having severe hearing loss, 8.9% having profound hearing loss, and 85.6% having complete hearing loss. The mean DMFT score was 2.5 ± 1.86, with no significant difference observed between males and females. The mean dmft score was 4.2 ± 3.12, with no significant difference between genders. However, there was a difference in the PI scores between males and females. Males presented a higher PI score of 2.6 ± 0.09 compared to 1.8 ± 0.08 for females. The overall mean PI was 2.1 ± 0.80. The mean GI was 1.5 ± 0.90, and no significant difference was observed between males and females. Spearman’s test identified a significant positive correlation between the severity of hearing impairment and both PI scores (p = 0.000) and GI scores (p = 0.000). Conversely, the severity of hearing impairment showed a weak positive correlation with both DMFT scores (p = 0.487) and dmft scores (p = 0.229), but these correlations were not statistically significant.

Conclusions

The connection between oral health and severe hearing impairment in children is significant and has potential implications. Pediatric healthcare providers, including dentists and audiologists, need to work collaboratively to monitor the oral and aural health of young patients.

## Introduction

Hearing impairment in children, whether occurring from birth or developing during childhood, involves a partial or complete inability to hear sounds, which can vary from mild to severe and may impact one or both ears [1]. This condition can be linked to factors such as genetic disorders, infections, loud noise exposure, or specific medications, all of which hinder the child’s capacity to perceive and comprehend sounds, including speech and environmental noise [2].

Hearing-impaired children may face challenges in communication, social interaction, academic performance, and emotional well-being, which can have a notable impact on their overall quality of life [3]. Beyond these challenges, oral health can also be significantly impacted, and studies have shown that children who are deaf or hard of hearing may experience poorer oral hygiene and have a higher prevalence of dental caries compared to their hearing peers [4].

The relationship between hearing impairment and oral health is an intriguing and interconnected aspect of overall well-being that merits attention and exploration. While these two sensory functions may seem unrelated at first glance, several underlying factors and commonalities highlight the intricate links between hearing impairment and oral health. People with severe hearing loss may face challenges in effectively communicating with dental care providers, which can lead to misunderstandings about oral care instructions and treatment plans. This can result in inadequate oral hygiene practices and neglect of dental issues [5].

Furthermore, individuals with severe hearing loss may be more prone to stress and anxiety [6], which can affect their oral health by increasing the risk of conditions such as gingival disease and dental caries. Stress and anxiety can weaken the immune system and contribute to inflammation in the body, including the gingiva [7].

Further, some medications used to manage hearing impairment can have side effects that impact oral health, such as antibiotics, chemotherapy drugs, and high doses of aspirin which are used to treat hearing problems; antihistamines, decongestants, and antidepressants which are used to manage the psychological condition associated with hearing impairment; and immune suppressants in cases of immune-related hearing disorders [8], all of which lead to dry mouth, taste disturbances, increased plaque accumulation, inflammation, and gum problems [9].

In addition to medication side effects and communication challenges, there are physical issues such as salivary flow, where some individuals with hearing impairment may experience changes in salivary flow due to stress or medication side effects. Reduced saliva production can lead to dry mouth, increasing the risk of dental plaque and gingivitis [6].

In addition, changes in the oral microbiome, influenced by factors such as stress or medication, could contribute to conditions such as gingivitis and plaque formation in individuals with hearing impairment [10,11]. Genetic predispositions that contribute to both hearing impairment and oral health issues could explain the observed correlation. Shared genetic pathways or susceptibility to certain conditions may underlie the co-occurrence of hearing loss severity and dental problems [12].

Understanding how these aspects intersect can provide valuable insights into the holistic care and management of individuals facing challenges in both domains. Therefore, this study aimed to investigate the relationship between the severity of hearing impairment and oral health in children with hearing disabilities and shed light on these interconnected health issues to promote better oral health outcomes and improve the quality of life for these children.

## Materials and methods

A cross-sectional study was conducted at the Institute of Special Education for the Hearing Impaired among children aged 6-12 years with hearing impairments during the academic year 2022. Rather than sampling, this study employed a total population approach, initially considering 110 children with hearing impairments, of which 20 were excluded, leading to the inclusion of 90 children with hearing impairments.

Parents of the participating children provided written and signed consent. Children were excluded from the study if they had other systemic diseases, had prolonged absences from school, were undergoing orthodontic treatment, or exhibited uncooperative behavior. The children participating in the study were of Syrian descent, aged 6-12 years, and met the study criteria.

Ethical approval was granted by the Directorate of Social Affairs and Labor in Damascus, and the study protocol received approval from the Ethics Committee of the Scientific Research and Postgraduate Board at Damascus University (approval number: UDDS-2649-02082021/SRC-1550).

The study protocol involved conducting pure-tone audiometry to assess the severity of hearing impairment in children [13]. Following the classification recommendations of the Global Burden of Disease (GBD) Expert Group on Hearing Loss, where the hearing level is defined as the better ear hearing threshold in decibels averaged over frequencies 0.5, 1, 2, and 4 kHz (dBHL). It has been classified into six categories of hearing impairment proposed by the GBD Expert Group as follows: mild (20-34 dBHL), moderate (35-49 dBHL), moderately severe (50-64 dBHL), severe (65-79 dBHL), profound (80-94 dBHL), complete (≥95 dBHL) [14].

An oral examination was conducted by the principal investigator using specialized equipment such as an explorer probe and a plain mouth mirror to evaluate caries, plaque accumulation, and gingivitis. The decayed, missing, and filled teeth (DMFT) and decayed, missing, and filled primary teeth (dmft) indices were evaluated, with the D component representing all teeth affected by caries or filled due to caries, the M component indicating missing teeth affected by untreated caries, and the F component including teeth filled without caries. For primary teeth, the calculation of the dmft index followed a similar approach [15].

The Silness-Löe plaque index (PI) was recorded as follows: 0 = no plaque visible in the gingival area, 1 = thin film of plaque adhering to the free gingival margin and nearby tooth surfaces, detectable with a probe, 2 = moderate accumulation of soft deposits in the gingival pocket, on the gingival margin, or adjacent tooth surfaces, visible to the naked eye, 3 = abundant soft matter within the gingival pocket or on the gingival margin and nearby tooth surfaces [16].

The gingival index (GI) by Löe and Silness was also documented: 0 = healthy gingiva, 1 = mild inflammation with slight color change and no bleeding on probing, 2 = moderate inflammation with redness and bleeding on probing, 3 = severe inflammation with marked redness and tendency for spontaneous bleeding [16].

During the examination, the children were seated and provided with a head-held light, disposable gloves, dental mirror, and explorer probes to examine for caries. Tongue depressors and cotton rolls were used to clear food residues and moisture that could obstruct the view of the teeth.

Statistical analysis included descriptive statistics. The chi-square test was used to identify significant differences between the two groups (male and female). Spearman’s test was used to determine the correlation between variables. Data analysis was performed using SPSS version 25.0 (IBM Corp., Armonk, NY, USA) with a significance level set at 5% (p < 0.05).

## Results

The study included 90 children aged 6-12 years with hearing impairment. Among them, 26 (28.9%) were in the 6-8-year age group, while 64 (71.1%) were in the 9-12-year age group. Regarding gender distribution, 58 (64%) were males, and 32 (36%) were females. The mean age was 9.7 ± 0.25 for males and 9.5 ± 0.30 for females. The severity of hearing impairment varied within the group: 5.6% had severe hearing loss (65-79.9 dB), 8.9% had profound hearing loss (80-94.9 dB), and 85.6% had complete hearing loss (≤95 dB) (Table 1).

**Table 1:** Descriptive statistics of the sample. HI = hearing impairment

Variables		Number	Percent (%)
Gender	Male	58	64%
	Female	32	36%
Age	6–8 years	26	28.9%
	9–12 years	64	71.1%
HI	severe hearing loss (65–79.9 dB)	5	5.6%
	profound hearing loss (80–94.9 dB)	8	8.9%
	complete hearing loss (≥95 dB)	77	85.6%

Female participants exhibited more severe hearing impairment compared to males (mean = 1 ± 0.01 vs. 0.96 ± 0.01, respectively) (p = 0.005) (Table *2*). About permanent teeth, the mean DMFT score was 2.5 ± 1.86, with no significant difference observed between males and females. For primary teeth, the mean dmft score was 4.2 ± 3.12, with no significant difference between genders. However, males displayed a higher PI score, indicating increased plaque accumulation, with scores of 2.6 ± 0.09 compared to 1.8 ± 0.08 for females (p = 0.032). The overall mean PI was 2.1 ± 0.80. On the other hand, the mean GI was 1.5 ± 0.90, with no significant difference between males and females. Further details are available in Table 2.

**Table 2:** Distribution of hearing impairments and dental indices according to gender. Significance of bold values, p < 0.05. DMFT = decay, missing, and filling teeth (permanent teeth); dmft = decay, missing, and filling teeth (primary teeth); PI = plaque index; GI = gingival index

Hearing impairments		Male		Female		Total		P-value
		Mean	SD	Mean	SD	Mean	SD	
		0.96	0.01	1	0.01	0.97	0.07	0.005
Dental Indices	DMFT	2.55	0.27	2.31	0.27	2.47	1.86	0.306
	dmft	4.12	0.39	4.66	0.61	4.17	3.12	0.166
	PI	2.59	0.09	1.76	0.08	2.05	0.80	0.032
	GI	1.43	0.12	1.69	0.15	1.52	0.90	0.719

Spearman’s test identified a significant positive correlation between the severity of hearing impairment and both PI scores (r = 0.458, p = 0.000), and GI scores (r = 0.419, p = 0.038). Conversely, the severity of hearing impairment showed a weak positive correlation with both DMFT scores (r = 0.074, p = 0.487), and dmft scores (r = 0.111, p = 0.229), although this relationship did not reach any statistical significance (Table 3).

**Table 3:** Relationship between hearing impairment and dental indices. **: Correlation is significant at the 0.01 level. Significance of bold values, p < 0.05. DMFT = decay, missing, and filling teeth (permanent teeth); dmft = decay, missing, and filling teeth (primary teeth); PI = plaque index; GI = gingival index

	Hearing impairment	
	Correlation coefficient	Significance (two-tailed)
DMFT	0.074	0.487
dmft	0.111	0.229
PI	0.458**	0.000
GI	0.419**	0.000

## Discussion

This study is considered the pioneering research endeavor to investigate the correlation between oral health and the severity of hearing impairment in Syrian children. Studying these relationships in children with hearing impairments is essential for holistic health understanding. It could enable early detection and intervention strategies, promote preventive measures, encourage interdisciplinary collaboration among healthcare professionals, and support educational programs for optimal health outcomes [17].

The study findings indicated a higher occurrence of dental caries among children with hearing impairment, possibly influenced by factors such as age, the severity of hearing loss, and inadequate education or awareness about oral hygiene practices [18]. This aligns with previous research by Alyami et al., which reported dental caries as a common issue affecting 65% of children with hearing impairments [19]. Interestingly, there was no significant difference between genders in terms of dental caries prevalence, consistent with the study by Sandeep et al. [20]. However, this contrasts with the finding of Ballal et al., who reported that male children had a higher mean DMFT compared to females among children with speech and hearing impairment in Ramapuram, Chennai [21].

Poor oral health, such as plaque buildup and gingivitis, was found in children with hearing impairment in this study. This may be linked to inadequate oral hygiene, unhealthy diet, limited parental awareness, and delays in receiving dental treatments. These children also faced challenges with postural control, motor skills, and quality of life, which could affect their ability to uphold good oral hygiene practices [22]. In addition to communication barriers that hinder understanding of oral hygiene instructions and expressing dental concerns, limited access to information and resources on oral health, sensory sensitivities that may make dental visits uncomfortable, a lack of specialized dental care providers experienced in working with special needs children, and challenges in developing and maintaining proper oral hygiene habits [4].

Significant differences were observed between males and females regarding plaque accumulation, while no significant difference was observed between genders in terms of GI. However, Jnaneswar et al reported differences in bleeding on probing and calculus between males and females in hearing-impaired children [23]. These discrepancies in PI between males and females may be attributed to a combination of biological factors, such as hormonal variations, behavioral factors related to oral hygiene practices, and environmental factors including access to dental care, socioeconomic status, and dietary habits [24].

The study revealed a significant positive correlation between the severity of hearing impairment in children and both PI and GI scores, indicating that as the severity of the children’s hearing impairment increased, there was a corresponding increase in the amount of plaque buildup on their teeth, making them more prone to developing gingivitis. This may be because severe hearing impairment imposes additional challenges, primarily manifested in increased communication problems, which are then followed by difficulty receiving information and avoiding visits to the dentist due to fear, anxiety, and limited access to preventive information related to proper care and nutrition [25]. The existence of common risk factors between auditory diseases and oral problems may exacerbate the situation, such as general health status and socioeconomic status [26].

This was consistent with a study by Khalaf et al., where a lower level of oral care was observed in individuals with severe-to-profound hearing impairment/deafness compared to those with mild-to-moderate hearing impairment [27]. In contrast, this contradicts the findings of a study by Shivakumar et al., who reported a decrease in the average values of the dental PI among deaf children aged 5-18 years, as the accumulation of dental plaque is affected by several factors that differ from one individual to another, including the level of awareness and knowledge of oral health practices, especially the method and frequency of tooth brushing [28]. On the other hand, it can be explained by the possible side effects of some medications that are more commonly used by those with severe hearing impairments [8], which lead to increased dental plaque accumulation and gingivitis [9].

Furthermore, a weak correlation was observed for both DMFT and dmft scores, which was not statistically significant. This means the link between hearing loss and increased cavity risk in children was not conclusively demonstrated by the data. Despite the indication of a higher incidence of dental caries in children with hearing impairments due to middle ear inflammation, this has been attributed to the higher rate of pathogens, particularly *Streptococcus mutans* [29,30].

It is clear that there is a bidirectional relationship between oral health and hearing in children. Addressing one issue can have a positive impact on the other. Pediatric healthcare providers, including dentists and audiologists, need to work collaboratively to monitor the oral and aural health of their young patients. Early intervention and treatment of either dental problems or hearing loss is crucial to prevent the other condition from developing.

During the study involving children with hearing impairments, several limitations were identified. These include the small sample size, the study being limited to a specific location, and the lack of investigation of the amount of knowledge and the amount of health awareness among the parents. Further research is still needed to fully elucidate the mechanisms by which poor oral health can directly contribute to hearing impairment in children. Longitudinal studies following children from a young age would provide valuable insights. Meanwhile, parents, educators, and clinicians need to recognize the strong connection between these two critical aspects of child health and development. Promoting good oral hygiene habits and providing timely access to dental and hearing care can go a long way in supporting the overall well-being of children.

## Conclusions

The connection between oral health and severe hearing impairment in children is significant and has potential implications. Pediatric healthcare providers should intervene early to address both oral and hearing health concerns. Timely treatment of dental or hearing issues is crucial to prevent complications and enhance overall well-being, including promoting good oral hygiene habits and ensuring access to dental and hearing care.
